# A novel origin for calcium selectivity

**DOI:** 10.7554/eLife.55216

**Published:** 2020-02-25

**Authors:** Esteban Suárez-Delgado, León D Islas

**Affiliations:** Department of Physiology, School of Medicine, UNAMMéxico CityMexico

**Keywords:** ion selectivity, ion channels, electrophysiology, calcium channel, Other

## Abstract

A native calcium ion channel has been identified in bacteria for the first time.

**Related research article** Shimomura T, Yonekawa Y, Nagura H, Tateyama M, Fujiyoshi Y, Irie K. 2020. A native prokaryotic voltage-dependent calcium channel with a novel selectivity filter sequence. *eLife* **9**:e52828. doi: 10.7554/eLife.52828

Articles in the popular media often show neurons and muscle cells launching miniature flashes of lightning when they are stimulated. In real cells, these processes are less glossy but still fascinating. Movements, thoughts, feelings, memories, sensations and more are made possible by ions moving in and out of cells through narrow pores in proteins called ion channels, which open and close in response to various stimuli (such as a change in voltage or membrane tension, or a molecule binding to the ion-channel protein). Ion channels are embedded in the cell membrane, and the ionic currents passing through them change the voltage across the membrane, creating an electrical signal that can be propagated to the interior of the cell or to other cells (Hille, 2001).

Like all other proteins, ion channels are the product of natural selection (Anderson and Greenberg, 2001), and we can learn about their evolution by comparing the genes that code for similar ion channels in different species (Moran and Zakon, 2014). An important property of an ion channel is its selectivity, which determines the types of ions that can pass through it. Sodium ion channels have been identified in bacteria and other prokaryotes, and some of these channels can be mutated to gain selectivity for calcium, but until recently no native calcium ion channels had been observed in prokaryotes.

Now, in eLife, Katsumasa Irie of Nagoya University and colleagues – including Takushi Shimomura as first author – report the first identification of a native prokaryotic calcium ion channel in *Meiothermus ruber*, a species of bacteria that lives in hot springs (Shimomura et al., 2020). They also propose a selectivity mechanism that is different from that found in calcium ion channels in eukaryotes.

By aligning multiple DNA sequences of sodium and calcium channels from prokaryotes, Shimomura et al. also reconstructed a phylogenetic tree to show how sodium and calcium channels in different species evolved from a common ancestor (Figure 1). This revealed a new branch of the phylogenetic tree containing what they have called ancestor-like sodium channels (which are homologous to contemporary sodium channels). The selectivity filters of the newly identified calcium channel and the ancestor-like sodium channels have sequences similar to the one predicted for an ancestor of a sodium channel found in bacteria (Liebeskind et al., 2013) and, remarkably, to the sequences found in a number of calcium channels from mammals.

**Figure 1.**
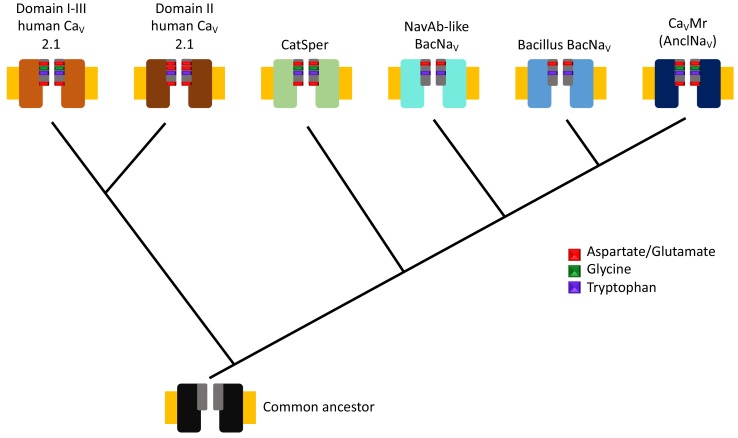
Evolutionary tree for calcium and sodium ion channels. The results of Shimomura et al. suggest that two types of human calcium channel domains (top left), human CatSper (a set of calcium channels found in sperm), the bacterial sodium channels homologous to the one found in *Arcobacter butzleri* (NavAb-like BacNavs), the bacterial sodium channels homologous to those from *Bacillus* species (*Bacillus* BacNavs), and the native calcium channel found in *M. ruber* (and also the ancestor-like sodium channels; see main text) have a common ancestor (bottom). The ion channels are represented by two identical subunits, and the selectivity filters are shown in grey with the most conserved residues shown in color (see guide). Note that the presence of glycine can be associated with calcium selectivity in both the human calcium channels and *M. ruber* calcium channels. The plasma membrane is represented in yellow.

The sequences for two homologues of the prokaryotic sodium channels were then used to synthesize the corresponding channels in mammalian and insect cells, so that their electrical properties could be measured. The channel based on *M. ruber* showed a high selectivity for ions with a charge of 2+: indeed, its selectivity for Ca^2+^ was ~200 times greater than its selectivity for Na^+^. It is generally thought that the selectivity of calcium channels is due to the presence of aspartates, which are negatively charged, in the selectivity filter (Catterall and Zheng, 2015). However, when the *M. ruber* calcium channel was mutated to eliminate an aspartate in this region, the channel retained most of its selectivity for Ca^2+^ ions.

On the other hand, the channel from *Plesiocystis pacifica*, a bacterial species that lives in soil, was three times more selective for Na^+^ ions than it was for Ca^2+^ ions. Moreover, the flow of Na^+^ ions through this channel could be blocked by high concentrations of extracellular Ca^2+^ ions. Furthermore, the blocking effect of Ca^2+^ could be enhanced by adding an alanine residue, which has no charge, to the selectivity filter, despite the presence of three negatively charged amino acids in this region.

These findings suggest that the selectivity of these two ion channels may not solely depend on the presence of negative charges in the filter.

The results from *M. ruber* and *P. pacifica* prompted Shimomura et al. to investigate the molecular basis of Ca^2+^ selectivity in these channels. They found that if the selectivity filter of the *M. ruber* calcium channel was mutated to be the same as the *P. pacifica* sodium channel, this new channel lost its Ca^2+^ selectivity and behaved like a non-selective channel. However, when the *P. pacifica* sodium channel was mutated to be the same as the *M. ruber* calcium channel, it demonstrated levels of calcium selectivity similar to those observed in *M. ruber*.

*M. ruber* and *P. pacifica* have different amino acids at positions 4 and 6 in their selectivity filters. Shimomura et al. found that changing the glycine at position 4 in *M. ruber* to a serine or an aspartate reduced the Ca^2+^ selectivity by a factor of almost 25, and wiped out any selectivity for ions with a single positive charge (such as Na^+^). The small and flexible glycine residue in position 4 would make the pore wider and facilitate the entry of ions with a charge of 2+, such as Ca^2+^. The conservation of this glycine residue in some eukaryotic calcium channels suggests that it might be part of a more general calcium-selectivity mechanism. Structural analysis of the new channels will shed light on this possibility.

Finally, the latest work indicates that voltage-dependent Ca^2+^ signaling is more ancient than previously thought, and the presence of calcium channels in prokaryotes suggests the possibility that Ca^2+^-selective channels in eukaryotes are not derived from sodium channels, as previously postulated. The new family of channels described by Shimomura et al. opens a tantalizing window into a vast evolutionary landscape that we are just beginning to grasp.
